# Night shift work and dietary behaviors: a comparative analysis of European night shift and day workers from the SHIFT2HEALTH online survey

**DOI:** 10.1186/s12937-026-01320-y

**Published:** 2026-04-07

**Authors:** Urte Klink, Edith J. M. Feskens, Desiree A. Lucassen, Monique H. Vingerhoeds, Sandra Haider, Kyriaki Papantoniou, Imke Matullat, Catalina Cuparencu, Anna Lena Aufschnaiter, Bianca Fuchs-Neuhold, Christina Höfler, Marlies Wallner, Katarzyna W. Binder-Olibrowska, Magdalena Agnieszka Wrzesińska, Heidi M. Lammers-van der Holst, Isabel Santonja, Katrin Scionti, Vanessa Schoissengeier, Karl-Heinz Wagner, Maria Wakolbinger, Eva Winzer, Meeke Ummels, Hannah Jilani

**Affiliations:** 1 University of Bremen, Institute for Public Health and Nursing Research, Bremen, Germany; 2 Wageningen University & Research, Division of Human Nutrition and Health, Wageningen, Netherlands; 3 Wageningen Food & Biobased Research, Wageningen, Netherlands; 4 Centre for Public Health, Department of Social and Preventive Medicine, Medical University of Vienna, Vienna, Austria; 5 Department of Epidemiology, Centre for Public Health, Medical University of Vienna, Vienna, Austria; 6 Barcelona Institute for Global Health (ISGlobal), Barcelona, Spain; 7 ttz Bremerhaven, Bremerhaven, Germany; 8 Department of Nutrition, Exercise and Sports, University of Copenhagen, Copenhagen, Denmark; 9 University of Applied Sciences FH JOANNEUM, Institute of Dietetics and Nutrition, Graz, Austria; 10 Department of Psychosocial Rehabilitation, Medical University of Lodz, Lodz, Poland; 11 Department of Public Health, Erasmus University Medical Center, Rotterdam, Netherlands; 12 Department of Nutritional Sciences, University of Vienna, Vienna, Austria; 13 Vienna Doctoral School of Pharmaceutical, Nutritional and Sport Sciences (PhaNuSpo), Vienna, Austria

**Keywords:** Night shift work, Diet, Food Intake, Food Preferences, Diet Surveys, Europe

## Abstract

**Background:**

Night shift workers are more likely to exhibit unfavorable dietary behaviors than day workers. However, these differences remain insufficiently explored within the European workforce. This study aims to examine differences in dietary behaviors (food intake frequency, eating frequency) by night shift exposure (history, frequency, duration) to investigate eating frequency, meal timing, and food choice determinants during night shifts, and to explore gender differences across Europe.

**Methods:**

Data were collected via an online survey (May 2024-January 2025) in eight countries (Austria, Germany, Denmark, Greece, Italy, the Netherlands, Poland, Spain). Participants self-reported sociodemographics, occupational sector, current work schedules, shift work history, lifestyle characteristics, and dietary behaviors. A shortened Food Frequency Questionnaire (FFQ) assessed dietary intake, alongside questions on eating rate and frequency on work and non-work-days. Current night shift workers additionally reported eating frequency, timing, and food choice determinants during night shifts. Analyses compared dietary behaviors by night shift exposure (current, former, vs. day worker). Among current night shift workers, associations with night shift frequency (nights/month) and duration (years) were examined. Secondary analyses were stratified by gender.

**Results:**

A total of 6,260 individuals were included (mean age 40.7, SD 10.7; 50.5% female). Overall, 60.4% were current night shift workers, 19.6% former night shift workers, and 20% day workers. Compared to day workers, current night shift workers reported significantly faster eating rates (OR = 1.21, 95% CI: 1.06–1.37), more frequent intake of sugar-sweetened (OR = 1.30, 95% CI: 1.14–1.48) and caffeinated beverages (OR = 1.14, 95% CI: 1.01–1.30), and lower fruit intake (OR = 0.87, 95% CI: 0.77–0.98). Higher monthly night shift load and longer duration of night work were associated with less favorable dietary patterns. Most current night shift workers reported one to two eating occasions per night, typically at the beginning or middle of their shift. Food choices were primarily driven by appetite, time, and food availability. Gender differences were observed only in food choice determinants.

**Conclusions:**

Night shift and day workers in Europe showed differences in dietary behaviors, particularly in sugar-sweetened beverage intake and eating rate. These findings highlight the need for targeted interventions to promote healthy eating among shift working populations.

**Supplementary Information:**

The online version contains supplementary material available at 10.1186/s12937-026-01320-y.

## Introduction

Working time outside of the usual hours 8 a.m. to 6 p.m. has become ubiquitous across economically developed nations [1]. Occupational sectors such as healthcare, manufacturing, and transportation routinely require non-standard working hours, which are performed in fixed morning, evening, night shifts, and/or rotating shift schedules [1, 2]. In 2023, 18% of all employees in the European Union (equivalent to nearly 35 million workers) were employed in shift work, with slightly more men (19%) than women (17%). This proportion has remained relatively stable over the last two decades [3].

Night shift workers experience misalignment between the body’s circadian rhythms and the environment due to their irregular working hours, which have been associated with various negative health outcomes [4, 5]. For instance, meta-analyses have shown that shift work is associated with an increased body mass index (BMI) and a higher risk of overweight and obesity [6, 7]. Additionally, night shift work has been associated with other non-communicable diseases such as cardiovascular diseases [8] and type 2 diabetes mellitus [9]. As current research suggests that lifestyle-related behaviors such as dietary behaviors may be a key pathway underlying the obesogenicity of night shift work [10], further investigations are needed to better understand the links between night shift work and these behaviors.

Food intake differs significantly in night and day workers. Compared to day workers, rotating night shift workers (e.g., nurses) consume fewer fruits and vegetables, but more confectionery, savory snacks, and sugar-sweetened beverages [11]. Systematic reviews by Souza et al. [12] and Clark et al. [13] have shown that night shift workers tend to have a higher 24-h energy intake, lower intake of nutrient-dense core foods, and greater intake of discretionary and unhealthy foods, including saturated fats and sugar-sweetened beverages [12, 13]. Additionally, gender-specific differences have previously been reported among shift workers [14–16]. Some aspects of the differences in dietary behaviors between shift workers and day workers remain unclear, especially regarding food group intake, where evidence is still limited. Moreover, existing studies often compare dietary behaviors of shift workers with different shift schedules (e.g., [15, 17, 18]) or only focus on a single occupational sector (e.g., [17–20]), rather than examining differences between night shift workers and day workers across multiple sectors.

Recent evidence suggests that night shift workers exhibit more unfavorable eating behaviors than day workers, including irregular meal patterns, frequent meal skipping, increased food intake at unconventional times, more frequent nighttime eating, and higher eating rates [12, 13, 21]. These behaviors have been associated with an increased risk of obesity [22–25], and may therefore contribute to unfavorable weight outcomes. However, evidence on eating frequency (i.e., the number of daily eating occasions) remains inconclusive [26–29], and we identified only one study that investigated eating rate among shift workers [21]. Additionally, evidence is scarce on eating behaviors during night shifts, particularly the frequency and timing of eating occasions and the factors influencing food choices, as only a few studies have examined these behaviors [16, 18, 30, 31].

In sum, dietary behaviors such as food intake, eating rate, eating frequency, and meal timing during night shifts may partly explain the comparatively higher risk of obesity observed among shift workers. Still, evidence remains limited, particularly across countries in Europe, as most studies on shift workers’ dietary behaviors have been conducted within individual countries [13], making it difficult to generalize findings across cultures. Additionally, investigations into dietary behaviors of shift workers have been conducted in small cohorts of around 500 participants [13], limiting the potential to capture the diversity of work patterns and contexts, improve statistical precision, and allow for more robust comparisons across groups. To date, no large, comprehensive Europe-wide assessment has examined how different work schedules relate to dietary behaviors. This is essential in order to deepen our understanding of the dietary mechanisms linking night shift work to obesity, which can ultimately inform targeted interventions to promote a healthier eating pattern in this target group.

Therefore, the primary aim of this study was to investigate differences in dietary behaviors between current and former night shift workers and day workers, including potential gender differences. Secondary aims were to explore the influence of night shift exposure (frequency and duration) on dietary behaviors among current night shift workers, and to investigate eating frequency, meal timing and factors determining food choices during the night shifts.

## Methods

### Study design

This analysis is based on data from a Europe-wide online survey conducted within the SHIFT2HEALTH research project, which assessed work schedules alongside dietary and other health-related behaviors to investigate individuals with varying levels of night shift exposure. SHIFT2HEALTH is an EU-Horizon funded collaborative study involving 15 academic and non-academic partners across seven European countries. Its goal is to advance the understanding of behavioral and physiological factors contributing to higher obesity rates among night shift workers (https://shift2health.eu/).

### Recruitment and participants

Participant recruitment was conducted in eight European countries (Austria, Denmark, Germany, Greece, Italy, the Netherlands, Poland, and Spain) and took place between May 2024 and January 2025 through social media platforms (e.g., LinkedIn, Facebook, Instagram), European Institute of Innovation and Technology (EIT) Health newsletters, company partnerships, labor associations, personal networks, and an agency specializing in social research. Eligible participants had to be 21 years or older, currently employed or self-employed, and working a minimum of 28 h per week. A total of 8,102 individuals participated in the online survey. Participation in the study was entirely voluntary. All participants provided online written informed consent prior to taking part. Only participants with information on all variables of interest were included in this analysis in order to conduct a complete case analysis.

### Questionnaire and measures

The survey was specifically developed for the SHIFT2HEALTH project and covered topics related to health and well-being, dietary behavior, physical activity, sleep behavior, and work schedules. It was available in eight languages (Danish, Dutch, English, German, Greek, Italian, Polish, Spanish), and included both validated and non-validated items. Specifically, the validated items were taken from instruments that have been previously developed and published by other research groups. References for the items relevant to this paper are provided in the corresponding sections. If a validated version of a questionnaire was not available in the required language, forward and backward translation and pre-testing was performed, to ensure accuracy [32]. The non-validated items were developed by the authors specifically for this survey to address research aspects not covered by existing validated instruments. Their development was guided by existing literature and expert consensus. The questionnaire metadata and full codebook for the SHIFT2HEALTH online survey can be accessed through the Zenodo repository [33].

Completing the full survey required approximately 15 to 20 min, and was fully anonymous. The survey was conducted using Research Electronic Data Capture (REDCap, https://project-redcap.org/), and the data was securely stored on a server at the University of Vienna.

#### Night shift work

No validated questionnaire exists that solely assesses shift work exposure. However, the survey questions had previously been used in other studies [34, 35], were informed by the expert consensus paper by Stevens et al., [36], which outlines key shift work domains to be captured in future research, and were developed by content expert.

Night shift exposure was assessed using the question “Have you ever worked night shifts (schedule including ≥ 3 h of work between midnight and 5 a.m.)? “ with response options (0) “No”, (1) “Yes, in the past”, and (2) “Yes, currently”. Accordingly, participants were classified as day workers, former night shift workers, and current night shift workers, respectively. Further, participants were asked to complete a form detailing their current work schedule, including start and end times and the number of shifts worked in a month. Both former and current night shift workers provided information on the duration of night shift work (total number of years). Current night shift workers are asked about their shift rotation patterns, and former night shift workers reported the number of years that had passed since their last night shift.

For the purpose of this analysis, individuals with very low night shift exposure were excluded: former night shift workers who had worked night shifts for less than one year in total and whose last night shift simultanously occurred more than 10 years ago (N = 69), as well as current night shift workers with less than six months of night shift exposure (N = 36).

#### Dietary behaviors

##### Food group intake

Food group intake frequencies were assessed using a subset of items adapted from the Rapid Prime Diet Quality Score (rPDQS) [37], with an additional question on caffeinated beverages. Participants were asked the following question: “Thinking back over the past month, how often have you eaten each of the following foods?” The food groups assessed included: (1) white bread, white rice, white pasta; (2) whole grain bread, brown rice, whole grain pasta, whole grain breakfast cereal, cold or cooked; (3) legumes and pulses like beans, lentils, chickpeas, tofu; (4) vegetables, fresh or cooked (e.g., carrots, string beans, spinach, kale, broccoli, cauliflower, cabbage—NOT potatoes); (5) whole fruits, fresh or cooked (e.g., apples, oranges, bananas, berries, grapes, melons), NOT juice; (6) soda, soft drinks, sports drinks without caffeine (not including diet drinks); and (7) coffee or other drinks containing caffeine or taurin or guaranas such as energy drinks.

For each food group, participants selected from five possible answers: (1) “less than once per week”, (2) “once per week”, (3) “2–4 times/week”, (4) “nearly daily or daily”, (5) “twice per day or more”.

The rPDQS uses a traffic light system informed by the 2021 American Heart Association dietary guidance and evidence linking food group intake to major diet-related diseases. Intake frequencies are categorized as “red,” “yellow,” or “green,” with “red” indicating suboptimal or unfavorable intake levels, and “green” indicating intake levels considered optimal or favorable [37]. Answer categories were collapsed accordingly. Caffeinated/stimulant beverages are presented as less than daily, nearly daily/daily, and twice or more times/day.

To improve readability of the text, the following abbreviated terms will be used henceforth: (1) refined grains, (2) whole grains, (3) legumes and pulses, (4) vegetables, (5) fruits, (6) sugar-sweetened beverages, and (7) caffeinated/stimulant beverages.

##### Eating rate

Eating rate was assessed by the question “How would you describe the speed at which you eat compared to others?” with the answer options (1) “very slow”, (2) “slow”, (3) “average”, (4) “fast”, (5) “very fast”. This question has previously been validated in a Dutch population [38].

##### Eating frequency

Eating frequency was assessed through three separate questions, asking participants how often they eat something (ranging from a small snack to a large meal) on work and work-free days, and, if applicable, during night shifts. For each question, the answer categories were (1) “1–2”, (2) “3–4”, (3) “5–6”, and (4) “7 or more”. Additionally, for eating frequency during night shifts, the answer category “0” (i.e., “I do not eat”) was provided.

##### Meal timing during night shifts

Current night shift workers who indicated that they eat something during a night shift were asked “How are the eating occasions usually divided over your night shift? “ to assess meal timing during night shifts. Answer categories read as follows: (1) “At the beginning of my shift”, (2) “In the middle of my night shift”, (3) “At the end of my night shift”, and (4) “It differs per shift”. Participants were asked to select all options that apply to them.

Given that eating frequency and meal timing was relevant to the overall aim of the project, but survey length constraints prevented the use of a comprehensive chrononutrition questionnaire, items were developed based on expert consensus.

##### Food choice determinants during night shifts

Current night shift workers who indicated that they eat something during night shifts were asked “What determines the kind of food you eat during night shifts?” to assess factors influencing food choices during night shifts. Answer options read as follows: (1) “My appetite,” (2) “My habits,” (3) “Time available,” (4) “Cost,” (5) “Food available,” (6) “Mood,” (7) “Energy level,” and (8) “Other”. Participants were asked to select all options that apply to them. This question was based on food choice determinants previously assessed by Waterhouse et al. [30], with additional response options capturing affective and physical influences (mood and energy level) on food choice added based on expert input and evidence from the literature [39].

#### Covariates

Age and gender were assessed as single items in the questionnaire. Due to very low number of responses, the gender categories “non-binary” (N = 16), “other” (N = 2), and “prefer not to say” (N = 2) were excluded from this analysis. BMI was calculated automatically using self-reported weight and height, defined as body weight (kg) divided by the square of height (m^2^).

Level of education was assessed according to the 2011 International Standard Classification of Education (ISCED) [40] with the question “What is your educational level (highest level achieved)?”. Response options were adapted to account for country-specific differences and included: (1) “basic education not completed” (ISCED 0–1), (2) “basic education” (ISCED 2), (3) “high school and vocational training” (ISCED 3), (4) “advanced training and college” (ISCED 4–5), (5) “university degrees and beyond” (ISCED 6–8), (6) “unknown/prefer not to say.”

Country of residence was assessed with the question “In which country do you currently live?” with the answer options Austria, Denmark, Estonia, Germany, Greece, Ireland, Italy, Latvia, Lithuania, the Netherlands, Poland, Romania, Spain, and Other. Participants from countries with very low number of respondents (Baltic countries, Ireland, Romania, Other) and missing entries were grouped into the category “Others.”

Participants were asked to indicate the occupational sector of their primary job, choosing from 15 work sectors categories derived from the Austrian Employment Service [41].

### Statistical analysis

All analyses were conducted in R version 4.4.2. Descriptive statistics are presented as means and standard deviations (SD) for continuous variables and frequencies (N, %) for categorical variables, stratified by day workers, former night shift workers, and current night shift workers. Descriptive statistics for eating behaviors during night shifts were stratified by gender.

Associations between night shift exposure and dietary behaviors (food group intake frequency, eating rate, and eating frequency) were examined using mixed-effects ordinal logistic regression models fitted with the clmm() function from the *ordinal* package [42]. Country of residence was included as a random intercept to account for clustering. The proportional odds assumption was evaluated graphically by inspecting cumulative probabilities across predictor categories.

Former and current night shift workers were compared with day workers (reference group). Food group intake frequency was analyzed using the original five ordinal response categories. Crude models are presented in the Supplementary Material. Model 1 was adjusted for age and gender; Model 2 was additionally adjusted for BMI, education level, and occupational sector. Covariates were selected based on prior evidence identifying them as potential confounders [3, 7, 43–49].

Effect modification by gender was assessed by including an interaction term between night shift exposure and gender (male/female) in Model 1 and comparing models with and without the interaction using likelihood ratio tests.

Additional analyses explored dietary behaviors according to night shift frequency (nights/month) and duration (years). Current night shift workers were categorized into tertiles of frequency and duration and compared with day workers; former night shift workers were excluded from these analyses. Participants who did not report night shifts (N = 1,069) were excluded from analyses of night shift frequency. Crude models are presented in the Supplementary Material.

Associations between eating behaviors during night shifts and gender (male/female) were assessed using the clmm() function from the R package ordinal [42] for ordinal (eating frequency during night shift) and binary outcomes (timing of eating occasions, determinants of food choice). Models were adjusted for age and included a random intercept for country.

Sensitivity analyses were conducted by excluding healthcare workers and by adjusting for employment in the healthcare sector; results remained materially unchanged.

A *p* value of less than 0.05 was considered to be significant. All confidence intervals (CI) are presented as 95% CI.

## Results

### Sociodemographic characteristics of the study population

A total of 8,102 individuals participated in the online survey, of which 1,842 participants had to be excluded due to implausible responses (N = 110), missing values in variables of interest (N = 1,607), low night shift exposure (former and current night shift workers, N = 105), and because gender analyses were restricted to participants identifying as female or male (other genders, N = 20). The final study population consisted of 6,260 participants, with 1,226 (19.6%) day workers, 1,250 (20%) former and 3,784 (60.4%) current night shift workers. The mean age of participants was 40.7 (SD 10.7) years, 50.5% were female, and 44.1% held a university degree. Average BMI was slightly higher in former and current night shift workers (26 (SD 5.3) kg/m^2^) compared to day workers (25.1 (SD 5.2) kg/m^2^). The sample included a large number of participants (52% of current night shift and 21% of day workers) from the health, social care and beauty sector (see Table 2 in Supplementary Materials for more details on occupational sectors). Participants reported an average working time of 39.2 (SD 6.2) hours per week, with current night shift workers reporting slightly longer hours (39.7) than day workers (38.2). Among current night shift workers who reported night shifts in the survey, 32% worked more than 8 night shifts per month, and 32.8% had worked night shifts for more than 12 years. Nearly half of former night shift workers had worked night shifts for one to five years. Among former night shift workers, the last night shift occurred on average 5.2 (SD 6.2) years ago (Table 1).

**Table 1:** Sociodemographic characteristics and night shift history by night shift exposure and for the total study population

	Day workers	Former night shift workers	Current night shift workers	Overall
	N = 1,226	N = 1,250	N = 3,784	N = 6,260
Gender N (%)				
Male	515 (42.0%)	645 (51.6%)	1937 (51.2%)	3097 (49.5%)
Female	711 (58.0%)	605 (48.4%)	1847 (48.8%)	3163 (50.5%)
Age (years) Mean (SD)	40.4 (10.5)	41.0 (10.5)	40.6 (10.7)	40.7 (10.7)
BMI (kg/m^2^) Mean (SD)	25.1 (5.23)	26.1 (5.2)	26.2 (5.4)	26.0 (5.32)
Level of education N (%)				
Basic education not completed	4 (0.3%)	12 (1.0%)	11 (0.3%)	27 (0.4%)
Basic education	61 (5.0%)	94 (7.5%)	243 (6.4%)	398 (6.4%)
High school and vocational training	311 (25.4%)	310 (24.8%)	922 (24.4%)	1543 (24.6%)
Advanced training and college	240 (19.6%)	323 (25.8%)	968 (25.6%)	1531 (24.5%)
University degrees and beyond	610 (49.8%)	511 (40.9%)	1640 (43.3%)	2761 (44.1%)
Health, social care, beauty work sector N (%)	256 (20.9%)	375 (30.0%)	1966 (52.0%)	2597 (41.5%)
Work hours per week Mean (SD)	38.2 (5.2)	38.9 (5.6)	39.7 (6.6)	39.2 (6.2)
Number of reported night shifts per month N (%) *				
1–5 night shifts/month			953 (35.1%)	
6–8 night shifts/month			894 (32.9%)	
> 8 night shifts/month			868 (32.0%)	
Total years of night shift work N (%)				
> 0.5—≤ 5 years			1,367 (36.1%)	
> 5—< 13 years			1,175 (31.0%)	
≥ 13 years			1,240 (32.8%)	
Total years of night shift work N (%)				
Less than one year		246 (19.9%)		
1–5 years		594 (48.1%)		
6–15 years		291 (23.6%)		
> 15 years		103 (8.4%)		
Years since last night shift Mean (SD)		5.2 (6.2)		

^*^Current night shift workers who did not report night shifts were excluded (*N* = 1,069)

Details on country-specific differences in population characteristics are provided in the Supplementary Materials (Table (suppl) 1).

### Night shift work and dietary behaviors

Table 2 provides information on dietary behaviors by night shift exposure. More details on food group intake frequency are available in the Supplementary Materials (Table (suppl) 3).

**Table 2:** Food group intake frequency, eating rate, and eating frequency by night shift exposure and in the total study population

	**Day workers**	**Former night shift workers**	**Current night shift workers**	**Overall**
	*N* = 1,226	*N* = 1,250	*N* = 3,784	*N* = 6,260
*Food group intake frequency*				
Refined grains				
Red (nearly daily/daily, twice or more times/day)	347 (28.3%)	384 (30.7%)	1174 (31.0%)	1905 (30.4%)
Yellow (2–4 times per week)	440 (35.9%)	449 (35.9%)	1393 (36.8%)	2282 (36.5%)
Green (less than once per week, once per week)	439 (35.8%)	417 (33.4%)	1217 (32.2%)	2073 (33.1%)
Whole grains				
Red (less than once per week, once per week)	472 (38.5%)	486 (38.9%)	1499 (39.6%)	2457 (39.2%)
Yellow (2–4 times per week)	376 (30.7%)	355 (28.4%)	1170 (30.9%)	1901 (30.4%)
Green (nearly daily/daily, twice or more times/day)	378 (30.8%)	409 (32.7%)	1115 (29.5%)	1902 (30.4%)
Legumes and pulses				
Red (less than once per week)	280 (22.8%)	278 (22.2%)	970 (25.6%)	1528 (24.4%)
Yellow (once per week, 2–4 times per week)	824 (67.2%)	802 (64.2%)	2330 (61.6%)	3956 (63.2%)
Green (nearly daily/daily, twice or more times/day)	122 (10.0%)	170 (13.6%)	484 (12.8%)	776 (12.4%)
Vegetables				
Red (less than once per week, once per week, 2–4 times per week)	704 (57.4%)	733 (58.6%)	2197 (58.1%)	3634 (58.1%)
Yellow (nearly daily or daily)	370 (30.2%)	392 (31.4%)	1215 (32.1%)	1977 (31.6%)
Green (twice or more times per day)	152 (12.4%)	125 (10.0%)	372 (9.8%)	649 (10.4%)
Fruits				
Red (less than once per week, once per week, 2–4 times per week)	601 (49.0%)	685 (54.8%)	2056 (54.3%)	3342 (53.4%)
Yellow (nearly daily or daily)	410 (33.4%)	392 (31.4%)	1210 (32.0%)	2012 (32.1%)
Green (twice or more times per day)	215 (17.5%)	173 (13.8%)	518 (13.7%)	906 (14.5%)
Sugar-sweetened beverages				
Red (nearly daily/daily, twice or more times/day)	176 (14.4%)	226 (18.1%)	678 (17.9%)	1080 (17.3%)
Yellow (once per week, 2–4 times per week)	517 (42.2%)	524 (41.9%)	1635 (43.2%)	2676 (42.7%)
Green (less than once per week)	533 (43.5%)	500 (40.0%)	1471 (38.9%)	2504 (40.0%)
Caffeinated/stimulant beverages				
Less than daily (Less than once per week, once per week, 2–4 times per week)	408 (33.3%)	395 (31.6%)	1176 (31.1%)	1979 (31.6%)
Nearly daily/daily	437 (35.6%)	390 (31.2%)	1114 (29.4%)	1941 (31.0%)
Twice or more times/day	381 (31.1%)	465 (37.2%)	1494 (39.5%)	2340 (37.4%)
Eating rate				
Very slow	44 (3.6%)	21 (1.7%)	66 (1.7%)	131 (2.1%)
Slow	174 (14.2%)	170 (13.6%)	491 (13.0%)	835 (13.3%)
Average	622 (50.7%)	636 (50.9%)	1702 (45.0%)	2960 (47.3%)
Fast	319 (26.0%)	337 (27.0%)	1204 (31.8%)	1860 (29.7%)
Very fast	67 (5.5%)	86 (6.9%)	321 (8.5%)	474 (7.6%)
Eating frequency on work-free days				
1–2	310 (25.3%)	295 (23.6%)	840 (22.2%)	1445 (23.1%)
3–4	654 (53.3%)	692 (55.4%)	2165 (57.2%)	3511 (56.1%)
5–6	235 (19.2%)	232 (18.6%)	709 (18.7%)	1176 (18.8%)
7 or more	27 (2.2%)	31 (2.5%)	70 (1.8%)	128 (2.0%)
Eating frequency on workdays				
1–2	379 (30.9%)	413 (33.0%)	1117 (29.5%)	1909 (30.5%)
3–4	616 (50.2%)	616 (49.3%)	1979 (52.3%)	3211 (51.3%)
5–6	212 (17.3%)	188 (15.0%)	621 (16.4%)	1021 (16.3%)
7 or more	19 (1.5%)	33 (2.6%)	67 (1.8%)	119 (1.9%)

Approximately 30.4% of participants consumed refined grains at least once per day, placing them in the “red” category of the traffic light system by Kronsteiner-Gicevic et al. [37]. Likewise, 39.2% consumed whole grains once per week or less, 24.4% consumed legumes and pulses less than once per week, and more than half reported eating fruits and vegetables fewer than four times per week. Additionally, 17.3% of participants consumed sugar-sweetened beverages at least once per day, also falling into the “red” category. Overall, a higher proportion of current and former night shift workers fell into the “red” category of the traffic light system across most food groups, except for legumes and pulses. The proportion of participants consuming caffeinated beverages at least twice a day was higher among current (39.5%) and former night shift workers (37.2%) compared to day workers (31.1%).

Most survey participants reported average (47.3%) or fast (29.7%) eating rates. A larger proportion of current night shift workers reported fast eating rates compared to day workers (31.8% vs. 26.0%, respectively).

Approximately half of all participants reported three to four eating occasions per day on both work and work-free days. Overall, eating frequency appeared to be higher on work-free days compared to work days.

Additionally, country differences were observed in food group intake frequencies, eating rate, and eating frequencies. Detailed country-specific findings are presented in the Supplementary Materials (Table (suppl) 4).

#### Associations between work schedule and dietary behaviors

Compared to day workers, current night shift workers reported less frequent fruit intake (Model 2: OR = 0.87, 95% CI: 0.77–0.98) and more frequent intake of refined grains (OR = 1.15, 95% CI: 1.03–1.30), sugar-sweetened (OR = 1.30, 95% CI: 1.14–1.48), and caffeinated beverages (OR = 1.14, 95% CI: 1.01–1.30), after adjusting for age, gender, BMI, education, occupational sector, and accounting for clustering by country (Table 3). Similarly, former night shift workers consumed caffeinated beverages more (OR = 1.17, 95% CI: 1.01–1.35), and fruits significantly less frequently (OR = 0.82, 95% CI: 0.71–0.94) than day workers.

**Table 3:** Ordinal logistic regression results comparing former and current night shift workers to day workers with respect to dietary behaviors

		**Day workers**	**Former night shift workers**			**Current night shift workers**		
		***N* = 1,226**	***N* = 1,250**			***N* = 3,784**		
		***ref***	**Odds Ratio**	**95% CI**		**Odds Ratio**	**95% CI**	
*Food group intake frequency*								
Refined grains	*Model 1*		**1.16**	**1.01**	**1.34**	**1.15**	**1.03**	**1.30**
	*Model 2*		1.14	0.99	1.32	**1.17**	**1.04**	**1.33**
Whole grains	*Model 1*		1.01	0.88	1.16	1.00	0.89	1.12
	*Model 2*		1.02	0.88	1.17	0.99	0.87	1.12
Legumes and pulses	*Model 1*		1.13	0.98	1.31	1.02	0.90	1.14
	*Model 2*		1.15	1.00	1.33	1.09	0.96	1.24
Vegetables	*Model 1*		1.00	0.87	1.15	0.98	0.87	1.10
	*Model 2*		1.05	0.90	1.21	1.00	0.88	1.14
Fruits	*Model 1*		**0.82**	**0.71**	**0.94**	**0.87**	**0.77**	**0.98**
	*Model 2*		**0.86**	**0.74**	**0.99**	**0.87**	**0.77**	**0.99**
Sugar-sweetened beverages	*Model 1*		1.14	0.99	1.32	**1.18**	**1.04**	**1.33**
	*Model 2*		1.13	0.98	1.31	**1.30**	**1.14**	**1.48**
Caffeinated/stimulant beverages	*Model 1*		**1.20**	**1.04**	**1.38**	**1.24**	**1.10**	**1.39**
	*Model 2*		**1.17**	**1.01**	**1.35**	**1.14**	**1.01**	**1.30**
Eating rate	*Model 1*		1.10	0.95	1.28	**1.35**	**1.20**	**1.53**
	*Model 2*		1.07	0.92	1.24	**1.21**	**1.06**	**1.37**
Eating frequency on work-free days	*Model 1*		1.05	0.89	1.22	**1.24**	**1.09**	**1.41**
	*Model 2*		1.09	0.93	1.27	**1.30**	**1.13**	**1.49**
Eating frequency on work days	*Model 1*		0.90	0.77	1.05	1.12	0.99	1.27
	*Model 2*		0.94	0.81	1.10	**1.17**	**1.02**	**1.33**

Odds ratios (OR) and 95% confidence intervals (CI) were estimated using mixed-effects ordinal logistic regression models comparing former and current night shift workers with day workers (reference group). Bold values indicate statistically significant results. Model 1: adjusted for gender and age, with country as random intercept; Model 2: Model 1 + adjusted for BMI, education, and occupational sector. Food group intake frequency was assessed on a 5-point scale from less than once per week to twice or more per day; eating rate on a 5-point scale from very slow to very fast; answer options for eating frequency on work and work-free days were 1–2, 3–4, 5–6, and ≥ 7 occasions per day

The analyses, based on ordinally scaled response categories, showed that current night shift workers had significantly higher eating rates compared to day workers (OR = 1.21, 95% CI: 1.06–1.37), while former night shift workers did not. In addition, current night shift workers also exhibited higher eating frequencies on work (OR = 1.17, 95% CI: 1.02–1.33) and work-free days (OR = 1.30, 95% CI: 1.13–1.49) compared to day workers.

The interaction between night shift exposure and gender (male/female), tested using a log-likelihood ratio test comparing Model 1 with and without the interaction, was not significant for any of the food groups or dietary behaviors (p > 0.05). Results are presented in the Supplementary Materials (Table (suppl) 8).

#### Associations between night shift exposure and dietary behaviors

Compared with day workers, current night shift workers exhibited distinct dietary behavioral patterns that varied by the intensity and duration of night work. Higher night work intensity (> 8 nights per month) was associated with less favorable dietary intake, including less frequent intake of fruits (OR = 0.75, 95% CI: 0.63–0.89), and more frequent intake of sugar-sweetened beverages (OR = 1.39, 95% CI: 1.18–1.65). In contrast, workers with less intense schedules (1–8 nights per month) reported significantly faster eating rates and more frequent eating occasions on work-free days (Table 4).

**Table 4:** Ordinal logistic regression results comparing current night shift workers of different levels of night work frequency (nights/month) to day workers in relation to dietary behaviors

		**Day workers**	Current night shift workers*									
			***5 night shifts/month***			***6–8 night shifts/month***			** > *8 night shifts/month***			***P* value for trend**
		**N = 1,230**	**N = 953**			**N = 894**			**N = 868**			
		***ref***	**Odds Ratio**	**95% CI**		**Odds Ratio**	**95% CI**		**Odds Ratio**	**95% CI**		
Food group intake frequency												
Refined grains	*Model 1*		1.17	1.00	1.38	1.14	0.97	1.34	1.16	0.98	1.36	0.081
	*Model 2*		**1.24**	**1.04**	**1.49**	1.17	0.98	1.39	1.15	0.97	1.37	0.173
Whole grains	*Model 1*		1.06	0.91	1.24	1.02	0.87	1.19	0.86	0.74	1.01	0.095
	*Model 2*		0.97	0.81	1.15	0.99	0.83	1.18	0.84	0.71	1.00	0.075
Legumes and pulses	*Model 1*		0.90	0.77	1.05	0.88	0.75	1.03	1.08	0.92	1.27	0.622
	*Model 2*		0.89	0.74	1.06	0.95	0.79	1.13	1.12	0.94	1.32	0.159
Vegetables	*Model 1*		1.17	0.99	1.37	1.10	0.93	1.29	**0.79**	**0.67**	**0.93**	**0.014**
	*Model 2*		1.14	0.95	1.36	1.15	0.96	1.38	0.85	0.72	1.01	0.087
Fruits	*Model 1*		1.04	0.89	1.22	1.01	0.86	1.19	**0.73**	**0.62**	**0.85**	** < 0.001**
	*Model 2*		0.97	0.81	1.16	1.03	0.86	1.22	**0.75**	**0.63**	**0.89**	**0.003**
Sugar-sweetened beverages	*Model 1*		0.87	0.74	1.03	1.00	0.85	1.18	**1.43**	**1.21**	**1.68**	** < 0.001**
	*Model 2*		0.89	0.74	1.07	0.94	0.79	1.13	**1.39**	**1.18**	**1.65**	** < 0.001**
Caffeinated/stimulant beverages	*Model 1*		**1.41**	**1.20**	**1.66**	**1.56**	**1.33**	**1.84**	1.04	0.89	1.22	0.115
	*Model 2*		**1.33**	**1.11**	**1.59**	**1.46**	**1.22**	**1.74**	0.99	0.84	1.17	0.758
Eating rate	*Model 1*		**1.39**	**1.18**	**1.64**	**1.43**	**1.21**	**1.68**	1.14	0.97	1.34	**0.035**
	*Model 2*		1.18	0.98	1.41	**1.24**	**1.03**	**1.48**	1.01	0.85	1.21	0.735
Eating frequency on work-free days	*Model 1*		**1.46**	**1.23**	**1.74**	**1.34**	**1.12**	**1.59**	1.15	0.97	1.37	0.087
	*Model 2*		**1.56**	**1.28**	**1.89**	**1.43**	**1.18**	**1.73**	**1.23**	**1.03**	**1.48**	0.059
Eating frequency on work days	*Model 1*		**1.21**	**1.02**	**1.43**	**1.19**	**1.01**	**1.41**	1.02	0.86	1.21	0.609
	*Model 2*		**1.22**	**1.01**	**1.47**	**1.25**	**1.04**	**1.50**	1.09	0.91	1.30	0.323

Odds ratios (OR) and 95% confidence intervals (CI) were estimated using mixed-effects ordinal logistic regression models comparing categories of night shift frequency (≤ 5, 6–8, and > 8 night shifts/month) with day workers (reference group). A linear trend across night shift frequency categories was tested by modeling the exposure variable as ordinal. Bold values indicate statistically significant results. Model 1: adjusted for gender and age, with country as random intercept; Model 2: Model 1 + adjusted for BMI, education, and occupational sector. Food group intake frequency was assessed on a 5-point scale from less than once per week to twice or more per day; eating rate on a 5-point scale from very slow to very fast; answer options for eating frequency on work and work-free days were 1-2, 3-4, 5-6, and ≥ 7 occasions per day

*Current night shift workers who did not report night shifts were excluded (N = 1,069)

Regarding the duration of night work, no consistent trends in food group intake were observed across categories. However, short night work experience (≤ 5 years) was associated with less frequent intake of fruits (OR = 0.85, 95% CI: 0.73–0.98) and more frequent intake of sugar-sweetened beverages (OR = 1.40, 95% CI: 1.20–1.63). Longer night work experience (≥ 13 years) was associated with more frequent consumption of caffeinated beverages (OR = 1.33, 95% CI: 1.12–1.57), suggesting a potential dose–response relationship. Additionally, workers with ≥ 13 years of night work experience reported less favorable eating behaviors, characterized by faster eating rates (OR = 1.51, 95% CI: 1.27–1.79) and higher eating frequencies on both work-free (OR = 1.57, 95% CI: 1.31–1.88) and work days (OR = 1.39, 95% CI: 1.16–1.66) (Table 5).

**Table 5:** Ordinal logistic regression results comparing current night shift workers of different levels of night work duration (years of night work) to day workers in relation to dietary behaviors

		**Day workers**	**Current night shift workers**									
			***5 years***			** > *5—*< *13 years***			** ≥ *13 years***			***P* value for trend**
		***N* = 1,226**	***N* = 1,367**			***N* = 1,175**			***N* = 1,240**			
		***ref***	**Odds Ratio**	**95% CI**		**Odds Ratio**	**95% CI**		**Odds Ratio**	**95% CI**		
Food group intake frequency												
Refined grains	*Model 1*		1.15	1.00	1.33	**1.26**	**1.08**	**1.46**	1.07	0.91	1.25	0.152
	*Model 2*		1.15	0.99	1.34	**1.25**	**1.07**	**1.46**	1.06	0.90	1.25	0.258
Whole grains	*Model 1*		0.99	0.86	1.14	1.05	0.91	1.22	0.97	0.83	1.13	0.950
	*Model 2*		0.98	0.84	1.13	1.03	0.89	1.21	0.98	0.83	1.15	0.988
Legumes and pulses	*Model 1*		1.16	1.00	1.34	1.03	0.89	1.19	0.87	0.75	1.02	0.082
	*Model 2*		**1.20**	**1.03**	**1.40**	1.09	0.93	1.27	0.96	0.81	1.13	0.507
Vegetables	*Model 1*		0.92	0.80	1.06	1.04	0.90	1.21	0.98	0.84	1.15	0.821
	*Model 2*		0.95	0.81	1.10	1.07	0.91	1.25	1.02	0.86	1.20	0.521
Fruits	*Model 1*		**0.86**	**0.74**	**0.99**	0.90	0.78	1.04	**0.85**	**0.73**	**1.00**	0.068
	*Model 2*		**0.85**	**0.73**	**0.98**	0.89	0.76	1.04	0.85	0.72	1.00	0.097
Sugar-sweetened beverages	*Model 1*		**1.33**	**1.15**	**1.54**	**1.23**	**1.06**	**1.42**	0.96	0.82	1.13	0.948
	*Model 2*		**1.40**	**1.20**	**1.63**	**1.29**	**1.10**	**1.51**	1.01	0.85	1.20	0.808
Caffeinated/stimulant beverages	*Model 1*		1.08	0.94	1.25	**1.24**	**1.07**	**1.43**	**1.46**	**1.25**	**1.71**	** < 0.001**
	*Model 2*		1.01	0.87	1.18	1.16	0.99	1.35	**1.33**	**1.12**	**1.57**	** < 0.001**
Eating rate	*Model 1*		1.03	0.89	1.19	**1.44**	**1.24**	**1.68**	**1.69**	**1.44**	**1.98**	** < 0.001**
	*Model 2*		0.96	0.82	1.12	**1.30**	**1.11**	**1.53**	**1.51**	**1.27**	**1.79**	** < 0.001**
Eating frequency on work-free days	*Model 1*		1.11	0.95	1.30	**1.23**	**1.04**	**1.44**	**1.44**	**1.22**	**1.70**	** < 0.001**
	*Model 2*		1.18	1.00	1.39	**1.30**	**1.10**	**1.54**	**1.57**	**1.31**	**1.88**	** < 0.001**
Eating frequency on work days	*Model 1*		0.96	0.82	1.11	**1.18**	**1.01**	**1.38**	**1.27**	**1.08**	**1.50**	**0.001**
	*Model 2*		1.03	0.87	1.21	**1.26**	**1.06**	**1.48**	**1.39**	**1.16**	**1.66**	** < 0.001**

Odds ratios (OR) and 95% confidence intervals (CI) were estimated using mixed-effects ordinal logistic regression models comparing categories of night work duration (≤ 5, > 5– < 13, and ≥ 13 years) with day workers (reference group). A linear trend across duration categories was tested by modeling night work duration as an ordinal variable. Bold values indicate statistically significant results. Model 1: adjusted for gender and age, with country as random intercept; Model 2: Model 1 + adjusted for BMI, education, and occupational sector. Food group intake frequency was assessed on a 5-point scale from less than once per week to twice or more per day; eating rate on a 5-point scale from very slow to very fast; answer options for eating frequency on work and work-free days were 1–2, 3–4, 5–6, and ≥ 7 occasions per day

### Eating behaviors during night shifts

Thirteen percent of night shift workers reported not eating anything during night shifts. Most night shift workers (55.9%) reported having one to two eating occasions per night shift, with slightly more women (26.2%) than men (21.1%) reporting three to four eating occasions (Table 6).

**Table 6:** Eating behaviors of current night shift workers, stratified by gender

	**Men**	**Women**	**Overall**
	**N = 1,937**	**N = 1,847**	**N = 3,784**
Eating frequency during night shifts			
0	311 (16.1%)	193 (10.4%)	504 (13.3%)
1–2	1096 (56.6%)	1021 (55.3%)	2117 (55.9%)
3–4	408 (21.1%)	484 (26.2%)	892 (23.6%)
5–6	93 (4.8%)	114 (6.2%)	207 (5.5%)
7 or more	29 (1.5%)	35 (1.9%)	64 (1.7%)
Timing of eating occasions during night shifts*	***N* = 1,626**	***N* = 1,654**	***N* = 3,280**
At the beginning of night shift	484 (29.8%)	473 (28.6%)	957 (29.2%)
In the middle of night shift	959 (59.0%)	897 (54.2%)	1856 (56.6%)
At the end of night shift	284 (17.5%)	363 (21.9%)	647 (19.7%)
It differs per shift	326 (20.0%)	496 (30.0%)	822 (25.1%)
Determinants of food choice during night shifts*			
My appetite	807 (49.6%)	852 (51.5%)	1659 (50.6%)
Time	598 (36.8%)	796 (48.1%)	1394 (42.5%)
Food availability	591 (36.3%)	702 (42.4%)	1293 (39.4%)
My habits	571 (35.1%)	491 (29.7%)	1062 (32.4%)
Energy level	285 (17.5%)	479 (29.0%)	764 (23.3%)
Mood	280 (17.2%)	452 (27.3%)	732 (22.3%)
Cost	243 (14.9%)	152 (9.2%)	395 (12.0%)
Other determinants	15 (0.9%)	28 (1.7%)	43 (1.3%)

*Items on timing of eating occasions and determinants of food choice allowed multiple responses and were analyzed separately

Most participants (56.5%) reported eating in the middle of their night shift. Smaller proportions reported eating at the beginning (29.2%) and/or at the end (19.7%) of their night shift, while 25.1% indicated that the timing of their eating differed between shifts. Additional explorations examined which individual and combined response options were most frequently selected. Results revealed that 38% of night shift workers selected only “in the middle of my night shift”, 19% selected only “it differs per shift”, and 13% selected only “at the beginning of my shift”. The most common combination was “at the beginning of my shift” and “in the middle of my shift” (6%).

Night shift workers commonly reported “my appetite” (50.6%), “time” (42.5%), and “food availability” (39.4%) as factors influencing their food choices during night shifts. Gender stratification revealed marked differences in several determinants, with women more often citing “time” (48.1% vs. 36.8%), “food availability” (42.4% vs. 36.3%), “mood” (27.3% vs. 17.2%), and “energy level” (29.0% vs. 17.5%) than men. Results showed that 9% of night shift workers cited their “habits” as the sole determinant of their food choices, followed by “my appetite” (9%) and “time” (7%). The most frequently reported combination was “my appetite” and “food availability” (4%).

#### Gender differences in eating behaviors during night shifts

Compared to men, women were significantly more likely to report that the timing of eating occasions during night shifts varies between shifts (OR = 1.42, 95% CI: 1.20–1.69), independent of age, and accounting for clustering by country, whereas no significant differences were observed in eating frequency or the timing of eating occasions. Additionally, women were more likely than men to identify “time” (OR = 1.53, 95% CI: 1.32–1.76), “energy level” (OR = 1.72, 95% CI: 1.44–2.04), and “mood” (OR = 1.60, 95% CI: 1.34–1.91) as determinants of food choice during night shifts, while they were less likely to report “my habits” (OR = 0.73, 95% CI: 0.62–0.85) and “cost” (OR = 0.58, 95% CI: 0.46–0.73) as determinants. Full results are presented in the Supplementary Materials (Table (suppl) 9).

## Discussion

Our analysis of dietary data from the SHIFT2HEALTH online survey revealed that, compared to day workers, current night shift workers reported faster eating rates, more frequent intake of refined grains, sugar-sweetened and caffeinated beverages, and lower fruit intake. Similar associations for former night shift workers suggest that certain dietary behaviors may persist even after cessation of night work. Patterns also indicated that greater exposure to night work—both in frequency and duration—was associated with poorer dietary intake and less favorable eating behaviors. Most night shift workers reported having one or two eating occasions during their shifts, typically at the beginning or middle of their shift. Food choices are largely influenced by appetite, time, and food availability. Gender differences only emerged in the factors influencing food choices during night shifts.

Overall, dietary intakes according to the traffic light system of the rPDQS by Kronsteiner-Gicevic et al. [37] appeared to be suboptimal among participants. Notably, a substantial proportion of participants fell into the “red” intake category for whole grains, fruits, and vegetables, indicating low intakes for these food groups. Poor dietary behaviors and low adherence to dietary guidelines are generally prevalent in European populations [50–52]. In our study, compared to day workers, both former and current night shift workers reported less frequent fruit consumption, while current night shift workers consumed refined grains more frequently, after adjusting for potential confounders. We also observed an increase in unfavorable food intake frequencies with an increase in number of monthly night shifts. Current evidence on the relationship between night shift exposure, fruit and refined grain intake appears inconclusive, as Souza et al. [12] reported inconsistent findings regarding the consumption of various food groups such as fruits, and breads and cereals. However, several studies not included in the review by Souza et al. [12] have reported lower intakes of fruit in night shift workers [11, 53, 54]. These differences may be attributed to differences in populations and the diverse methodological approaches employed across studies to assess dietary intake, including variations in data collection tools, reporting periods, and participant instructions. Given the non-significant findings for other food groups in this study and significant findings found in other shift-working populations [55], further research should focus on identifying contextual and occupational factors, such as shift patterns, job type, and cultural dietary norms, that may influence the intake of specific food groups among shift and day workers.

In the present study we observed lower intake of sugar-sweetened beverages than previously reported for high-income countries, including European countries [56]. This difference may partly reflect differences in beverage definitions across studies, as our assessment was restricted to uncaffeinated sugar-sweetened beverages. Current night shift workers consumed sugar-sweetened beverages more frequently than day workers, which is in line with previous studies [11, 12, 53]. Additionally, higher consumption of sugar-sweetened beverages among shift workers has been associated with increased workload [57] and higher work shift intensity [58], findings that align with our observation of a positive dose–response relationship with the number of night shifts per month. An increased intake of sweetened beverages among shift workers may present an important risk factor for higher body weight and other chronic diseases such as type 2 diabetes and metabolic syndrome [59, 60].

Daily intake of caffeinated beverages such as tea, coffee and energy drinks was common across the study population. Former and current night shift workers were more likely to report consuming such beverages two or more times per day, compared to day workers. This aligns with findings indicating higher caffeine consumption among nurses working night shifts [55]. Moreover, caffeinated beverage consumption increased with years of night shift work. Night shift workers have previously reported using caffeinated beverages to stay awake during their shifts [61, 62], while an increased consumption may also be attributable to a disrupted circadian rhythm [63]. Although the health benefits of moderate coffee consumption (1–4 cups per day) have been demonstrated [64], caffeine intake impairs subsequent sleep efficiency and duration [65], which may contribute to increased fatigue [66], potentially leading to a cycle of sleep disruption and caffeine use disorders. Therefore, monitoring and managing individual caffeine intake has been proposed as a strategy to improve sleep quality among shift workers [67].

Evidence on differences in eating rates between shift and day workers is limited, with only Nanri et al. [21] examining this association. While they reported higher eating rates among shift workers, results were not statistically significant. In our study, current night shift workers had significantly higher eating rates compared to day workers, and these rates increased with the number of years spent working night shifts. To our knowledge, this is the first study to present a significant association between shift work and eating rate, after adjusting for potential confounders. This finding is particularly important as faster eating has been linked to higher BMI and obesity [23, 38], conditions to which shift working populations are at elevated risk [6, 7]. Faster eating may increase obesity risk by enabling greater energy intake before satiety signals emerge [68, 69] and disrupting secretion of appetite hormones that regulate fullness [70].

Our analyses indicated that current night shift workers have more eating occasions on work-free days compared to day workers. No significant associations were observed for work days. However, number of eating occasions increased with years of night shift work. In systematic reviews by Souza et al. [12] and Clark et al. [13] evidence on differences in eating frequency between night shift and day workers, as well as across different shift types among shift workers were evaluated. While two of their included studies reported a higher eating frequency in shift workers compared to morning shift or day workers [26, 27], one study found a lower meal frequency in shift workers [28], and another observed no differences between shift and day workers [29]. Notably, these studies were conducted in different occupational sectors and used varying methodologies, such as 24-h dietary recalls and questionnaires, possibly contributing to inconsistent results across studies. Nevertheless, investigating eating frequency in night shift workers remains relevant, given its potential effect on weight status [22].

As previous studies have reported gender-differences in dietary behaviors among night shift workers [14–16], we additionally examined whether these differences vary by night shift exposure by including interaction terms between gender and shift work in the regression model. No significant gender-differences were observed, independent of age, suggesting that gender does not modify the association between night shift exposure and dietary behaviors in our sample.

We observed similar associations in dietary behaviors among current and former night shift workers compared to day workers, suggesting that certain dietary behaviors may persist even after cessation of night shift work. Previous studies have shown that sleep problems can persist after leaving night shift work [35] and metabolic syndrome is more prevalent among former male shift workers than current day workers [71], indicating that night shift work may have lasting effects on health-related behaviors. However, to our knowledge, no previous studies have specificially examined dietary behaviors in former night shift workers, highlighting a gap that warrants further research.

Moreover, we examined whether night shift frequency (nights/month) and duration (years of night work) is associated with dietary behaviors among current night shift workers compared to day workers. Patterns across exposure levels were not consistent, and due to the cross-sectional nature of this research, findings should be considered exploratory and hypothesis-generating only. Night shift frequency was associated with less favorable food intake frequencies for fruits, refined grains, and sugar-sweetened beverages. By contrast, longer night shift experience (in years) showed more pronounced associations with eating behaviors, notably faster eating rate, higher eating frequency, as well as greater intake frequency of caffeinated beverages. This suggests that the two exposure measures capture different risk profiles for dietary behaviors, with night shift duration showing stronger associations with eating behaviors and night shift frequency showing more variable associations with food intake. Such dose–response associations have also been investigated by other authors [61, 72, 73], however, both the methodological approaches and the findings vary across studies.

Most current night shift workers reported having one to two eating occasions per night shift, with meals typically occurring at the beginning or in the middle of the shift, suggesting these eating occasions predominantly happen in the first half of the shift. Generally, investigations into food intake during night shifts have revealed alterations, particularly in timing and type of food consumed [16, 18, 30, 31, 74]. For instance, a study by Kosmadopoulos et al. [18] examining the distribution of meal events and meal timing of caloric intake on night shifts revealed a later eating schedule and an erratic pattern of food consumption throughout the night. However, circular mean time of caloric intake occurred at around the start of a night shift [18], aligning with the findings presented in this study.

Current night shift workers most frequently mentioned appetite, time, and food availability as their main determinants of food choice during night shifts. Similar findings have been found by other authors [30, 75], indicating that food choices during night shifts are guided by situational factors and immediate physiological needs, rather than by more internal or personal factors. This suggests that effective interventions should aim to improve the availability and accessibility of healthy food options during night shifts, while also considering the unique time and appetite constraints faced by night shift workers.

Gender differences in eating behaviors during night shifts were evident in our sample. Women were more likely than men to report that the timing of eating occasions varied across shifts, and their food choices were more often driven by time constraints, energy level, and mood rather than habits and cost. Although we did not find direct evidence of gender-specific differences in food choices during night shifts, it is worth noting that women generally carry greater responsibilities at home than men [76], which may contribute to increased time pressure and help explain why their eating behaviors are more influenced by time constraints and energy levels.

### Strengths and limitations

The study holds several strengths. It is based on a large dataset comprising both shift and non-shift workers representing different European regions. To the best of our knowledge, no comparable dataset is currently available on work schedules and health-related behaviors across countries. Using predefined categories of night shift work alongside detailed information on shift schedules (e.g., frequency and duration) offers valuable insights into different patterns of night shift exposure. Validated questionnaire items were used to assess food intake frequency and eating rate, enhancing the reliability and comparability of the findings.

Several limitations of this study need to be acknowledged. The primary limitation concerns the nature of the study sample, which is not representative for the population of night shift and day workers. For instance, the sample included a larger proportion of individuals with higher levels of education in comparison to the EU population average [77]. This over-representation may be explained by the large number of health care workers who participated in the survey, as they often hold applied university degrees in many countries. Nonetheless, regression analyses adjusted for level of education and occupational sector revealed robust associations. The survey included several non-validated items, particularly regarding work schedules, eating frequency, and eating behaviors during night shifts, which may affect measurement validity and comparability with other studies. However, the items were developed based on relevant literature and expert input, and rigorous data cleaning was conducted. It is noted that food intake frequency does not provide information on the quantities consumed, which would have required a different dietary assessment method and was outside of the scope of this study. However, the rPDQS developed by Kronsteiner et al. [37] is a validated brief diet quality screener, capable of identifying clinically relevant food intake frequencies. In total, 1,600 participants had to be excluded due to missing data, potentially introducing selection bias. Lastly, all data were self-reported, and the cross-sectional nature of the survey precludes causal interpretations of the observed associations.

## Conclusions

Night shift workers reported significantly higher consumption of refined grains, sugar-sweetened and caffeinated beverages, and lower fruit intake compared to day workers. Current night shift workers reported significantly faster eating rates and more frequent eating occasions on work-free days compared to day workers, cumulatively indicating more unfavorable dietary behaviors in this population. Additionally, dose–response associations indicated less favorable dietary intake with higher monthly night shift load and less favorable eating behaviors with increasing years of night shift experience. Gender differences in dietary behaviors were only observed for eating behaviors during night shifts, particularly with regard to food choice determinants. While further research is still needed to clarify the associations between night shift work, dietary behaviors and adverse health outcomes, the present findings already indicate a potential benefit of targeted intervention strategies aimed at promoting healthier dietary behaviors in shift-working populations.

## Supplementary Information

Supplementary Material 1.

## Data Availability

The Codebook and Questionnaire Metadata for the SHIFT2HEALTH Online Survey are available in the Zenodo repository (https://doi.org/10.5281/zenodo.17423133).
