# Supplementary material for: Night shift work and dietary behaviors: a comparative analysis of European night shift and day workers from the SHIFT2HEALTH online survey
Source: Nutr J. 2026 Apr 7;25:43. doi: 10.1186/s12937-026-01320-y (PMC13067742; doi:10.1186/s12937-026-01320-y)
Supplement: Supplementary file 1 — Supplementary Material 1. [file 12937_2026_1320_MOESM1_ESM.docx]

# Supplementary Materials

Table (suppl) 1: Sociodemographic characteristics of study population by country and for the total study population

|  | Austria | Denmark | Germany | Greece | Italy | Poland | Spain | The Netherlands | Others^1^ | Overall |
| --- | --- | --- | --- | --- | --- | --- | --- | --- | --- | --- |
|  | N=982 | N=643 | N=790 | N=739 | N=604 | N=810 | N=725 | N=809 | N=155 | N=6,260 |
| Gender N (%) |  |  |  |  |  |  |  |  |  |  |
| Men | 331 (33.6%) | 337 (52.4%) | 417 (52.8%) | 470 (63.6%) | 390 (64.6%) | 433 (53.5%) | 398 (55.0%) | 250 (30.9%) | 71 (45.8%) | 3097 (49.5%) |
| Women | 655 (66.4%) | 306 (47.6%) | 373 (47.2%) | 269 (36.4%) | 214 (35.4%) | 377 (46.5%) | 326 (45.0%) | 559 (69.1%) | 84 (54.2%) | 3163 (50.5%) |
| Age (years) Mean (SD) | 34.5 (10.1) | 42.2 (10.4) | 42.1 (11.1) | 41.3 (9.01) | 43.8 (10.2) | 41.2 (9.65) | 43.2 (9.72) | 39.8 (11.5) | 40.1 (10.4) | 40.7 (10.7) |
| BMI (kg/m^2^) Mean (SD) | 25.8 (5.58) | 25.5 (5.96) | 27.3 (5.79) | 26.5 (5.05) | 24.8 (4.30) | 26.2 (5.33) | 25.0 (4.34) | 26.1 (5.26) | 26.1 (5.76) | 26.0 (5.32) |
| Level of education N (%) | |  |  |  |  |  |  |  |  |  |
| Basic education not completed | 3 (0.3%) | 9 (1.4%) | 3 (0.4%) | 0 (0%) | 1 (0.2%) | 3 (0.4%) | 7 (1.0%) | 0 (0%) | 1 (0.6%) | 27 (0.4%) |
| Basic education | 38 (3.9%) | 33 (5.1%) | 207 (26.2%) | 7 (0.9%) | 29 (4.8%) | 18 (2.2%) | 52 (7.2%) | 5 (0.6%) | 9 (5.8%) | 398 (6.4%) |
| High school and vocational training | 255 (25.9%) | 127 (19.8%) | 302 (38.2%) | 176 (23.8%) | 216 (35.8%) | 93 (11.5%) | 199 (27.5%) | 138 (17.1%) | 37 (23.9%) | 1543 (24.6%) |
| Advanced training and college | 258 (26.2%) | 183 (28.5%) | 78 (9.9%) | 192 (26.0%) | 83 (13.7%) | 315 (38.9%) | 148 (20.4%) | 236 (29.2%) | 38 (24.5%) | 1531 (24.5%) |
| University degrees and beyond | 432 (43.8%) | 291 (45.3%) | 200 (25.3%) | 364 (49.3%) | 275 (45.5%) | 381 (47.0%) | 318 (43.9%) | 430 (53.2%) | 70 (45.2%) | 2761 (44.1%) |
| Work sector N (%) |  |  |  |  |  |  |  |  |  |  |
| Construction, ancillary building trades, wood, building technology | 15 (1.5%) | 37 (5.8%) | 13 (1.6%) | 21 (2.8%) | 13 (2.2%) | 43 (5.3%) | 37 (5.1%) | 11 (1.4%) | 3 (1.9%) | 193 (3.1%) |
| Mining, raw materials, glass, ceramics, stone | 9 (0.9%) | 3 (0.5%) | 22 (2.8%) | 9 (1.2%) | 5 (0.8%) | 16 (2.0%) | 4 (0.6%) | 8 (1.0%) | 2 (1.3%) | 78 (1.2%) |
| Office, marketing, finance, law, security | 66 (6.7%) | 83 (12.9%) | 69 (8.7%) | 40 (5.4%) | 47 (7.8%) | 53 (6.5%) | 64 (8.8%) | 51 (6.3%) | 7 (4.5%) | 480 (7.7%) |
| Chemistry, biotechnology, food, plastics | 29 (2.9%) | 18 (2.8%) | 46 (5.8%) | 20 (2.7%) | 18 (3.0%) | 78 (9.6%) | 11 (1.5%) | 43 (5.3%) | 7 (4.5%) | 270 (4.3%) |
| Electrical engineering, electronics, tele-communications, IT | 25 (2.5%) | 92 (14.3%) | 45 (5.7%) | 29 (3.9%) | 38 (6.3%) | 33 (4.1%) | 39 (5.4%) | 21 (2.6%) | 11 (7.1%) | 333 (5.3%) |
| Trade, logistics, transport | 57 (5.8%) | 57 (8.9%) | 66 (8.4%) | 60 (8.1%) | 43 (7.1%) | 108 (13.3%) | 64 (8.8%) | 40 (4.9%) | 15 (9.7%) | 510 (8.1%) |
| Agriculture, horticulture, forestry | 4 (0.4%) | 14 (2.2%) | 4 (0.5%) | 25 (3.4%) | 11 (1.8%) | 13 (1.6%) | 13 (1.8%) | 5 (0.6%) | 4 (2.6%) | 93 (1.5%) |
| Mechanical engineering, automotive, metal | 20 (2.0%) | 34 (5.3%) | 25 (3.2%) | 18 (2.4%) | 15 (2.5%) | 48 (5.9%) | 39 (5.4%) | 11 (1.4%) | 4 (2.6%) | 214 (3.4%) |
| Media, graphics, design, printing, art, handicrafts | 1 (0.1%) | 15 (2.3%) | 3 (0.4%) | 11 (1.5%) | 8 (1.3%) | 13 (1.6%) | 11 (1.5%) | 5 (0.6%) | 2 (1.3%) | 69 (1.1%) |
| Cleaning, housekeeping, semiskilled and unskilled occupations | 8 (0.8%) | 5 (0.8%) | 9 (1.1%) | 8 (1.1%) | 20 (3.3%) | 32 (4.0%) | 27 (3.7%) | 6 (0.7%) | 3 (1.9%) | 118 (1.9%) |
| Health, social work, beauty care | 660 (66.9%) | 206 (32.0%) | 378 (47.8%) | 228 (30.9%) | 200 (33.1%) | 131 (16.2%) | 241 (33.3%) | 497 (61.4%) | 56 (36.1%) | 2597 (41.5%) |
| Textiles and clothing, fashion, leather | 5 (0.5%) | 5 (0.8%) | 3 (0.4%) | 9 (1.2%) | 7 (1.2%) | 11 (1.4%) | 9 (1.2%) | 3 (0.4%) | 0 (0%) | 52 (0.8%) |
| Tourism, catering, leisure | 27 (2.7%) | 10 (1.6%) | 12 (1.5%) | 105 (14.2%) | 31 (5.1%) | 17 (2.1%) | 44 (6.1%) | 14 (1.7%) | 13 (8.4%) | 273 (4.4%) |
| Environment | 1 (0.1%) | 2 (0.3%) | 1 (0.1%) | 6 (0.8%) | 3 (0.5%) | 6 (0.7%) | 5 (0.7%) | 0 (0%) | 0 (0%) | 24 (0.4%) |
| Science, education, research and development | 11 (1.1%) | 22 (3.4%) | 3 (0.4%) | 44 (6.0%) | 23 (3.8%) | 78 (9.6%) | 28 (3.9%) | 36 (4.5%) | 7 (4.5%) | 252 (4.0%) |
| Other | 48 (4.9%) | 40 (6.2%) | 91 (11.5%) | 106 (14.3%) | 122 (20.2%) | 130 (16.0%) | 88 (12.2%) | 58 (7.2%) | 21 (13.5%) | 704 (11.2%) |
| Work hours per week Mean (SD) | 39.9 (6.29) | 39.8 (7.82) | 39.2 (5.18) | 40.8 (6.16) | 38.7 (5.04) | 41.6 (5.59) | 39.3 (5.25) | 34.6 (5.02) | 40.4 (6.38) | 39.2 (6.18) |
| Night shift experience N (%) | |  |  |  |  |  |  |  |  |  |
| Day worker | 112 (11.4%) | 134 (20.8%) | 120 (15.2%) | 156 (21.1%) | 131 (21.7%) | 203 (25.1%) | 161 (22.2%) | 162 (20.0%) | 47 (30.3%) | 1226 (19.6%) |
| Former night shift worker | 118 (12.0%) | 113 (17.6%) | 164 (20.8%) | 181 (24.5%) | 77 (12.7%) | 188 (23.2%) | 156 (21.5%) | 204 (25.2%) | 49 (31.6%) | 1250 (20.0%) |
| Current night shift worker | 756 (76.7%) | 396 (61.6%) | 506 (64.1%) | 402 (54.4%) | 396 (65.6%) | 419 (51.7%) | 407 (56.2%) | 443 (54.8%) | 59 (38.1%) | 3784 (60.4%) |

^1^ Other countries include Ireland (N=6), Lithuania (N=1), Romania (N=1), other countries (N=44), and missings (N=103).

Table (suppl) 2: Work sector by night shift exposure and total sample.

|  | Day worker | Former night shift worker | Current night shift worker | Overall |
| --- | --- | --- | --- | --- |
|  | **N=1226** | **N=1250** | **N=3784** | **N=6260** |
| Work sector N (%) |  |  |  |  |
| Construction, ancillary building trades, wood, building technology | 54 (4.4%) | 70 (5.6%) | 69 (1.8%) | 193 (3.1%) |
| Mining, raw materials, glass, ceramics, stone | 9 (0.7%) | 15 (1.2%) | 54 (1.4%) | 78 (1.2%) |
| Office, marketing, finance, law, security | 165 (13.5%) | 119 (9.5%) | 196 (5.2%) | 480 (7.7%) |
| Chemistry, biotechnology, food, plastics | 30 (2.4%) | 45 (3.6%) | 195 (5.2%) | 270 (4.3%) |
| Electrical engineering, electronics, telecommunications, IT | 93 (7.6%) | 78 (6.2%) | 162 (4.3%) | 333 (5.3%) |
| Trade, logistics, transport | 121 (9.9%) | 124 (9.9%) | 265 (7.0%) | 510 (8.1%) |
| Agriculture, horticulture, forestry | 33 (2.7%) | 39 (3.1%) | 21 (0.6%) | 93 (1.5%) |
| Mechanical engineering, automotive, metal | 48 (3.9%) | 44 (3.5%) | 122 (3.2%) | 214 (3.4%) |
| Media, graphics, design, printing, art, handicrafts | 15 (1.2%) | 17 (1.4%) | 37 (1.0%) | 69 (1.1%) |
| Cleaning, housekeeping, semiskilled and unskilled occupations | 31 (2.5%) | 34 (2.7%) | 53 (1.4%) | 118 (1.9%) |
| Health, social work, beauty care | 256 (20.9%) | 375 (30.0%) | 1966 (52.0%) | 2597 (41.5%) |
| Textiles and clothing, fashion, leather | 20 (1.6%) | 14 (1.1%) | 18 (0.5%) | 52 (0.8%) |
| Tourism, catering, leisure | 66 (5.4%) | 78 (6.2%) | 129 (3.4%) | 273 (4.4%) |
| Environment | 6 (0.5%) | 8 (0.6%) | 10 (0.3%) | 24 (0.4%) |
| Science, education, research and development | 140 (11.4%) | 60 (4.8%) | 52 (1.4%) | 252 (4.0%) |
| Other | 139 (11.3%) | 130 (10.4%) | 435 (11.5%) | 704 (11.2%) |

Table (suppl) 3: Intake frequencies of food groups, stratified by night shift exposure and in the total sample

|  | Day worker | Former night shift worker | Current night shift worker | Overall |
| --- | --- | --- | --- | --- |
|  | **N=1226** | **N=1250** | **N=3784** | **N=6260** |
| Refined grains |  |  |  |  |
| Less than once per week | 166 (13.5%) | 151 (12.1%) | 432 (11.4%) | 749 (12.0%) |
| Once per week | 273 (22.3%) | 266 (21.3%) | 785 (20.7%) | 1324 (21.2%) |
| 2-4 times per week | 440 (35.9%) | 449 (35.9%) | 1393 (36.8%) | 2282 (36.5%) |
| Nearly daily or daily | 278 (22.7%) | 320 (25.6%) | 935 (24.7%) | 1533 (24.5%) |
| Twice per day or more | 69 (5.6%) | 64 (5.1%) | 239 (6.3%) | 372 (5.9%) |
| Whole grains |  |  |  |  |
| Less than once per week | 244 (19.9%) | 238 (19.0%) | 755 (20.0%) | 1237 (19.8%) |
| Once per week | 228 (18.6%) | 248 (19.8%) | 744 (19.7%) | 1220 (19.5%) |
| 2-4 times per week | 376 (30.7%) | 355 (28.4%) | 1170 (30.9%) | 1901 (30.4%) |
| Nearly daily or daily | 283 (23.1%) | 329 (26.3%) | 905 (23.9%) | 1517 (24.2%) |
| Twice per day or more | 95 (7.7%) | 80 (6.4%) | 210 (5.5%) | 385 (6.2%) |
| Legumes and pulses |  |  |  |  |
| Less than once per week | 280 (22.8%) | 278 (22.2%) | 970 (25.6%) | 1528 (24.4%) |
| Once per week | 405 (33.0%) | 405 (32.4%) | 1202 (31.8%) | 2012 (32.1%) |
| 2-4 times per week | 419 (34.2%) | 397 (31.8%) | 1128 (29.8%) | 1944 (31.1%) |
| Nearly daily or daily | 103 (8.4%) | 145 (11.6%) | 389 (10.3%) | 637 (10.2%) |
| Twice per day or more | 19 (1.5%) | 25 (2.0%) | 95 (2.5%) | 139 (2.2%) |
| Vegetables |  |  |  |  |
| Less than once per week | 61 (5.0%) | 62 (5.0%) | 225 (5.9%) | 348 (5.6%) |
| Once per week | 205 (16.7%) | 217 (17.4%) | 665 (17.6%) | 1087 (17.4%) |
| 2-4 times per week | 438 (35.7%) | 454 (36.3%) | 1307 (34.5%) | 2199 (35.1%) |
| Nearly daily or daily | 370 (30.2%) | 392 (31.4%) | 1215 (32.1%) | 1977 (31.6%) |
| Twice per day or more | 152 (12.4%) | 125 (10.0%) | 372 (9.8%) | 649 (10.4%) |
| Fruits |  |  |  |  |
| Less than once per week | 81 (6.6%) | 111 (8.9%) | 297 (7.8%) | 489 (7.8%) |
| Once per week | 172 (14.0%) | 212 (17.0%) | 650 (17.2%) | 1034 (16.5%) |
| 2-4 times per week | 348 (28.4%) | 362 (29.0%) | 1109 (29.3%) | 1819 (29.1%) |
| Nearly daily or daily | 410 (33.4%) | 392 (31.4%) | 1210 (32.0%) | 2012 (32.1%) |
| Twice per day or more | 215 (17.5%) | 173 (13.8%) | 518 (13.7%) | 906 (14.5%) |
| Sugar-sweetened beverages | | | |  |
| Less than once per week | 533 (43.5%) | 500 (40.0%) | 1471 (38.9%) | 2504 (40.0%) |
| Once per week | 266 (21.7%) | 250 (20.0%) | 805 (21.3%) | 1321 (21.1%) |
| 2-4 times per week | 251 (20.5%) | 274 (21.9%) | 830 (21.9%) | 1355 (21.6%) |
| Nearly daily or daily | 141 (11.5%) | 172 (13.8%) | 492 (13.0%) | 805 (12.9%) |
| Twice per day or more | 35 (2.9%) | 54 (4.3%) | 186 (4.9%) | 275 (4.4%) |
| Caffeinated/stimulant beverages |  |  |  |  |
| Less than once per week | 154 (12.6%) | 145 (11.6%) | 440 (11.6%) | 739 (11.8%) |
| Once per week | 99 (8.1%) | 97 (7.8%) | 262 (6.9%) | 458 (7.3%) |
| 2-4 times per week | 155 (12.6%) | 153 (12.2%) | 474 (12.5%) | 782 (12.5%) |
| Nearly daily or daily | 437 (35.6%) | 390 (31.2%) | 1114 (29.4%) | 1941 (31.0%) |
| Twice per day or more | 381 (31.1%) | 465 (37.2%) | 1494 (39.5%) | 2340 (37.4%) |

Table (suppl) 4: Food group intake frequency, eating speed, and eating frequency by country and in the total study sample

|  | Austria | Denmark | Germany | Greece | Italy | Poland | Spain | The Netherlands | Others^1^ | Overall |
| --- | --- | --- | --- | --- | --- | --- | --- | --- | --- | --- |
|  | N=982 | N=643 | N=790 | N=739 | N=604 | N=810 | N=725 | N=809 | N=155 | N=6,260 |
| *Food group intake frequency* | |  |  |  |  |  |  |  |  |  |
| Refined grains |  |  |  |  |  |  |  |  |  |  |
| Red (nearly daily/daily, twice or more times/day) | 290 (29.4%) | 161 (25.0%) | 183 (23.2%) | 242 (32.7%) | 235 (38.9%) | 417 (51.5%) | 245 (33.8%) | 87 (10.8%) | 45 (29.0%) | 1905 (30.4%) |
| Yellow (2-4 times per week) | 455 (46.1%) | 237 (36.9%) | 345 (43.7%) | 287 (38.8%) | 213 (35.3%) | 201 (24.8%) | 297 (41.0%) | 194 (24.0%) | 53 (34.2%) | 2282 (36.5%) |
| Green (less than once per week, once per week) | 241 (24.4%) | 245 (38.1%) | 262 (33.2%) | 210 (28.4%) | 156 (25.8%) | 192 (23.7%) | 182 (25.1%) | 528 (65.3%) | 57 (36.8%) | 2073 (33.1%) |
| Whole grains |  |  |  |  |  |  |  |  |  |  |
| Red (less than once per week, once per week) | 415 (42.1%) | 244 (37.9%) | 344 (43.5%) | 333 (45.1%) | 312 (51.7%) | 290 (35.8%) | 311 (43.0%) | 143 (17.7%) | 65 (41.9%) | 2457 (39.2%) |
| Yellow (2-4 times per week) | 352 (35.7%) | 203 (31.6%) | 233 (29.5%) | 231 (31.3%) | 162 (26.8%) | 255 (31.5%) | 232 (32.0%) | 186 (23.0%) | 47 (30.3%) | 1901 (30.4%) |
| Green (nearly daily/daily, twice or more times/day) | 219 (22.2%) | 196 (30.5%) | 213 (27.0%) | 175 (23.7%) | 130 (21.5%) | 265 (32.7%) | 181 (25.0%) | 480 (59.3%) | 43 (27.7%) | 1902 (30.4%) |
| Legumes and pulses |  |  |  |  |  |  |  |  |  |  |
| Red (less than once per week) | 312 (31.6%) | 183 (28.5%) | 291 (36.8%) | 50 (6.8%) | 70 (11.6%) | 325 (40.1%) | 44 (6.1%) | 222 (27.4%) | 31 (20.0%) | 1528 (24.4%) |
| Yellow (once per week, 2-4 times per week) | 555 (56.3%) | 334 (51.9%) | 427 (54.1%) | 577 (78.1%) | 475 (78.6%) | 397 (49.0%) | 585 (80.8%) | 499 (61.7%) | 107 (69.0%) | 3956 (63.2%) |
| Green (nearly daily/daily, twice or more times/day) | 119 (12.1%) | 126 (19.6%) | 72 (9.1%) | 112 (15.2%) | 59 (9.8%) | 88 (10.9%) | 95 (13.1%) | 88 (10.9%) | 17 (11.0%) | 776 (12.4%) |
| Vegetables |  |  |  |  |  |  |  |  |  |  |
| Red (less than once per week, once per week, 2-4 times per week) | 555 (56.3%) | 383 (59.6%) | 514 (65.1%) | 561 (75.9%) | 358 (59.3%) | 440 (54.3%) | 483 (66.7%) | 248 (30.7%) | 92 (59.4%) | 3634 (58.1%) |
| Yellow (nearly daily or daily) | 320 (32.5%) | 168 (26.1%) | 222 (28.1%) | 143 (19.4%) | 183 (30.3%) | 249 (30.7%) | 201 (27.8%) | 450 (55.6%) | 41 (26.5%) | 1977 (31.6%) |
| Green (twice or more times per day) | 111 (11.3%) | 92 (14.3%) | 54 (6.8%) | 35 (4.7%) | 63 (10.4%) | 121 (14.9%) | 40 (5.5%) | 111 (13.7%) | 22 (14.2%) | 649 (10.4%) |
| Fruits |  |  |  |  |  |  |  |  |  |  |
| Red (less than once per week, once per week, 2-4 times per week) | 600 (60.9%) | 372 (57.9%) | 478 (60.5%) | 466 (63.1%) | 286 (47.4%) | 416 (51.4%) | 337 (46.5%) | 299 (37.0%) | 88 (56.8%) | 3342 (53.4%) |
| Yellow (nearly daily or daily) | 268 (27.2%) | 180 (28.0%) | 234 (29.6%) | 201 (27.2%) | 221 (36.6%) | 269 (33.2%) | 277 (38.3%) | 322 (39.8%) | 40 (25.8%) | 2012 (32.1%) |
| Green (twice or more times per day) | 118 (12.0%) | 91 (14.2%) | 78 (9.9%) | 72 (9.7%) | 97 (16.1%) | 125 (15.4%) | 110 (15.2%) | 188 (23.2%) | 27 (17.4%) | 906 (14.5%) |
| Sugar-sweetened beverages | |  |  |  |  |  |  |  |  |  |
| Red (nearly daily/daily, twice or more times/day) | 148 (15.0%) | 164 (25.5%) | 186 (23.5%) | 106 (14.3%) | 47 (7.8%) | 187 (23.1%) | 104 (14.4%) | 120 (14.8%) | 18 (11.6%) | 1080 (17.3%) |
| Yellow (once per week, 2-4 times per week) | 454 (46.0%) | 325 (50.5%) | 302 (38.2%) | 372 (50.3%) | 232 (38.4%) | 342 (42.2%) | 311 (43.0%) | 261 (32.3%) | 77 (49.7%) | 2676 (42.7%) |
| Green (less than once per week) | 384 (38.9%) | 154 (24.0%) | 302 (38.2%) | 261 (35.3%) | 325 (53.8%) | 281 (34.7%) | 309 (42.7%) | 428 (52.9%) | 60 (38.7%) | 2504 (40.0%) |
| Caffeinated/stimulant beverages | |  |  |  |  |  |  |  |  |  |
| Less than once per week, once per week, 2-4 times per week | 279 (28.3%) | 298 (46.3%) | 254 (32.2%) | 219 (29.6%) | 175 (29.0%) | 198 (24.4%) | 237 (32.7%) | 266 (32.9%) | 53 (34.2%) | 1979 (31.6%) |
| Nearly daily or daily | 221 (22.4%) | 189 (29.4%) | 223 (28.2%) | 297 (40.2%) | 195 (32.3%) | 268 (33.1%) | 267 (36.9%) | 233 (28.8%) | 48 (31.0%) | 1941 (31.0%) |
| Twice or more times per day | 486 (49.3%) | 156 (24.3%) | 313 (39.6%) | 223 (30.2%) | 234 (38.7%) | 344 (42.5%) | 220 (30.4%) | 310 (38.3%) | 54 (34.8%) | 2340 (37.4%) |
| Eating rate |  |  |  |  |  |  |  |  |  |  |
| Very slow | 37 (3.8%) | 8 (1.2%) | 12 (1.5%) | 15 (2.0%) | 14 (2.3%) | 19 (2.3%) | 10 (1.4%) | 13 (1.6%) | 3 (1.9%) | 131 (2.1%) |
| Slow | 151 (15.3%) | 74 (11.5%) | 126 (15.9%) | 125 (16.9%) | 73 (12.1%) | 94 (11.6%) | 83 (11.5%) | 89 (11.0%) | 20 (12.9%) | 835 (13.3%) |
| Average | 368 (37.3%) | 352 (54.7%) | 349 (44.2%) | 330 (44.7%) | 320 (53.0%) | 447 (55.2%) | 322 (44.5%) | 408 (50.4%) | 64 (41.3%) | 2960 (47.3%) |
| Fast | 323 (32.8%) | 158 (24.6%) | 239 (30.3%) | 212 (28.7%) | 160 (26.5%) | 205 (25.3%) | 248 (34.3%) | 260 (32.1%) | 55 (35.5%) | 1860 (29.7%) |
| Very fast | 107 (10.9%) | 51 (7.9%) | 64 (8.1%) | 57 (7.7%) | 37 (6.1%) | 45 (5.6%) | 61 (8.4%) | 39 (4.8%) | 13 (8.4%) | 474 (7.6%) |
| Eating frequency on work-free days | |  |  |  |  |  |  |  |  |  |
| 1-2 | 220 (22.3%) | 123 (19.1%) | 245 (31.0%) | 201 (27.2%) | 259 (42.9%) | 95 (11.7%) | 210 (29.0%) | 58 (7.2%) | 34 (21.9%) | 1445 (23.1%) |
| 3-4 | 656 (66.5%) | 377 (58.6%) | 476 (60.3%) | 434 (58.7%) | 267 (44.2%) | 497 (61.4%) | 400 (55.2%) | 315 (38.9%) | 89 (57.4%) | 3511 (56.1%) |
| 5-6 | 106 (10.8%) | 128 (19.9%) | 65 (8.2%) | 98 (13.3%) | 74 (12.3%) | 199 (24.6%) | 105 (14.5%) | 374 (46.2%) | 27 (17.4%) | 1176 (18.8%) |
| 7 or more | 4 (0.4%) | 15 (2.3%) | 4 (0.5%) | 6 (0.8%) | 4 (0.7%) | 19 (2.3%) | 9 (1.2%) | 62 (7.7%) | 5 (3.2%) | 128 (2.0%) |
| Eating frequency on workdays | |  |  |  |  |  |  |  |  |  |
| 1-2 | 320 (32.5%) | 158 (24.6%) | 344 (43.5%) | 283 (38.3%) | 289 (47.8%) | 153 (18.9%) | 230 (31.8%) | 84 (10.4%) | 48 (31.0%) | 1909 (30.5%) |
| 3-4 | 491 (49.8%) | 367 (57.1%) | 383 (48.5%) | 354 (47.9%) | 254 (42.1%) | 505 (62.3%) | 399 (55.1%) | 383 (47.3%) | 75 (48.4%) | 3211 (51.3%) |
| 5-6 | 156 (15.8%) | 100 (15.6%) | 62 (7.8%) | 93 (12.6%) | 56 (9.3%) | 140 (17.3%) | 87 (12.0%) | 300 (37.1%) | 27 (17.4%) | 1021 (16.3%) |
| 7 or more | 19 (1.9%) | 18 (2.8%) | 1 (0.1%) | 9 (1.2%) | 5 (0.8%) | 12 (1.5%) | 8 (1.1%) | 42 (5.2%) | 5 (3.2%) | 119 (1.9%) |

^1^ Other countries include Ireland (N=6), Lithuania (N=1), Romania (N=1), other countries (N=44), and missings (N=103).

Table (suppl) 5: Results of the crude ordinal logistic regression comparing dietary behaviors of former and current night shift workers with those of day workers.

|  |  | Day workers | Former night shift workers | | | Current night shift workers | | |
| --- | --- | --- | --- | --- | --- | --- | --- | --- |
|  |  | **N=1,226** | **N=1,250** | | | **N=3,784** | | |
|  |  | ***ref.*** | **Odds Ratio** | **95% CI** | | **Odds Ratio** | **95% CI** | |
| *Food group intake frequency* | |  |  |  |  |  |  |  |
| Refined grains |  |  | **1.18** | **1.02** | **1.36** | **1.17** | **1.04** | **1.31** |
| Whole grains |  |  | 0.99 | 0.86 | 1.14 | 0.98 | 0.87 | 1.10 |
| Legumes and pulses |  |  | 1.13 | 0.98 | 1.30 | 1.01 | 0.90 | 1.14 |
| Vegetables |  |  | 0.93 | 0.80 | 1.07 | 0.91 | 0.81 | 1.03 |
| Fruits |  |  | **0.78** | **0.67** | **0.90** | **0.83** | **0.73** | **0.93** |
| Sugar-sweetened beverages |  |  | **1.21** | **1.05** | **1.40** | **1.24** | **1.10** | **1.40** |
| Caffeinated/ stimulant beverages |  |  | **1.18** | **1.03** | **1.36** | **1.23** | **1.10** | **1.38** |
| Eating rate |  |  | 1.15 | 0.99 | 1.33 | **1.41** | **1.25** | **1.59** |
| Eating frequency on work-free days |  |  | 1.00 | 0.86 | 1.17 | **1.18** | **1.04** | **1.34** |
| Eating frequency on work days |  |  | 0.88 | 0.76 | 1.03 | 1.09 | 0.96 | 1.23 |

*Odds ratios (OR) and 95% confidence intervals (CI) were estimated using mixed-effects ordinal logistic regression models with random intercepts for country, comparing former and current night shift workers with day workers (reference group) .Food group intake frequency was assessed on a 5-point scale from less than once per week to twice or more per day; eating rate on a 5-point scale from very slow to very fast; answer options for eating frequency on work and work-free days were 1–2, 3–4, 5–6, and ≥7 occasions per day.*

Table (suppl) 6: Crude ordinal logistic regression model comparing dietary behaviors of current night shift workers, stratified by night work frequency (nights/month), with those of day workers.

|  |  | Day workers | Current night shift workers* | | | | | | | | |  |
| --- | --- | --- | --- | --- | --- | --- | --- | --- | --- | --- | --- | --- |
|  |  |  | ***≤5 night shifts/month*** | | | ***6 – 8 night shifts/month*** | | | ***>8 night shifts/month*** | | | ***P* value for trend** |
|  |  | **N=1,230** | **N=953** | | | **N=894** | | | **N=868** | | |  |
|  |  | ***ref.*** | **Odds Ratio** | **95% CI** | | **Odds Ratio** | **95% CI** | | **Odds Ratio** | **95% CI** | |  |
| Food group intake frequency |  | | | | | | | | | | |  |
| Refined grains |  |  | 1.14 | 0.97 | 1.34 | 1.16 | 0.99 | 1.37 | **1.19** | **1.01** | **1.40** | **0.027** |
| Whole grains |  |  | 1.06 | 0.91 | 1.24 | 0.99 | 0.85 | 1.16 | 0.85 | 0.72 | 0.99 | **0.045** |
| Legumes and pulses |  |  | 0.89 | 0.76 | 1.04 | 0.87 | 0.74 | 1.02 | 1.08 | 0.92 | 1.26 | 0.655 |
| Vegetables |  |  | **1.20** | **1.02** | **1.40** | 0.98 | 0.84 | 1.16 | **0.72** | **0.61** | **0.84** | **<0.001** |
| Fruits |  |  | 1.08 | 0.92 | 1.26 | 0.93 | 0.79 | 1.09 | **0.67** | **0.57** | **0.78** | **<0.001** |
| Sugar-sweetened beverages |  |  | **0.83** | **0.71** | **0.98** | 1.10 | 0.94 | 1.29 | **1.57** | **1.34** | **1.84** | **<0.001** |
| Caffeinated/ stimulant beverages |  |  | **1.47** | **1.25** | **1.72** | **1.56** | **1.33** | **1.83** | 1.02 | 0.88 | 1.20 | 0.187 |
| Eating rate |  |  | **1.37** | **1.16** | **1.61** | **1.53** | **1.30** | **1.80** | **1.21** | **1.03** | **1.43** | **0.002** |
| Eating frequency on work-free days |  |  | **1.44** | **1.21** | **1.71** | **1.24** | **1.05** | **1.48** | 1.07 | 0.90 | 1.27 | 0.465 |
| Eating frequency on work days |  |  | **1.20** | **1.01** | **1.42** | 1.14 | 0.96 | 1.35 | 0.98 | 0.83 | 1.16 | 0.953 |

*Odds ratios (OR) and 95% confidence intervals (CI) were estimated using mixed-effects ordinal logistic regression models with random intercepts for country, comparing categories of night shift frequency (≤5, 6–8, and >8 night shifts/month) with day workers (reference group). A linear trend across night shift frequency categories was tested by modeling the exposure variable as ordinal. Food group intake frequency was assessed on a 5-point scale from less than once per week to twice or more per day; eating rate on a 5-point scale from very slow to very fast; answer options for eating frequency on work and work-free days were 1–2, 3–4, 5–6, and ≥7 occasions per day.*

Table (suppl) 7: Crude ordinal logistic regression models comparing dietary behaviors of current night shift workers, stratified by duration of night work (years), with those of day workers.

|  |  | Day workers | Current night shift workers | | | | | | | | |  |
| --- | --- | --- | --- | --- | --- | --- | --- | --- | --- | --- | --- | --- |
|  |  |  | ***≤5 years*** | | | ***>5 - <13 years*** | | | ***≥13 years*** | | | ***P* value for trend** |
|  |  | **N=1,226** | **N=1,367** | | | **N=1,175** | | | **N=1,240** | | |  |
|  |  | ***ref.*** | **Odds Ratio** | **95% CI** | | **Odds Ratio** | **95% CI** | | **Odds Ratio** | **95% CI** | |  |
| Food group intake frequency |  | | | | | | | | | | |  |
| Refined grains |  |  | **1.21** | **1.05** | **1.39** | **1.29** | **1.12** | **1.50** | 1.02 | 0.88 | 1.18 | 0.658 |
| Whole grains |  |  | 0.99 | 0.86 | 1.14 | 1.03 | 0.89 | 1.20 | **0.93** | **0.80** | **1.07** | 0.426 |
| Legumes and pulses |  |  | **1.18** | **1.03** | **1.36** | 1.03 | 0.89 | 1.19 | **0.84** | **0.73** | **0.97** | **0.004** |
| Vegetables |  |  | 0.91 | 0.79 | 1.05 | 0.97 | 0.84 | 1.13 | **0.86** | **0.74** | **0.99** | 0.085 |
| Fruits |  |  | 0.81 | 0.70 | 0.93 | 0.84 | 0.72 | 0.97 | **0.83** | **0.72** | **0.96** | **0.031** |
| Sugar-sweetened beverages |  |  | **1.43** | **1.24** | **1.65** | **1.33** | **1.15** | **1.54** | 0.99 | 0.86 | 1.15 | 0.674 |
| Caffeinated/ stimulant beverages |  |  | 0.96 | 0.84 | 1.11 | **1.18** | **1.02** | **1.37** | **1.69** | **1.46** | **1.95** | **<0.001** |
| Eating rate |  |  | 1.08 | 0.93 | 1.25 | **1.51** | **1.30** | **1.76** | **1.72** | **1.48** | **1.99** | **<0.001** |
| Eating frequency on work-free days |  |  | **1.20** | **1.03** | **1.40** | **1.20** | **1.02** | **1.41** | 1.13 | 0.97 | 1.32 | 0.169 |
| Eating frequency on work days |  |  | 1.03 | 0.88 | 1.19 | **1.17** | **1.00** | **1.37** | 1.06 | 0.91 | 1.23 | 0.227 |

*Odds ratios (OR) and 95% confidence intervals (CI) were estimated using mixed-effects ordinal logistic regression models with random intercepts for country, comparing categories of night work duration (≤5, >5–<13, and ≥13 years) with day workers (reference group). A linear trend across duration categories was tested by modeling night work duration as an ordinal variable. Food group intake frequency was assessed on a 5-point scale from less than once per week to twice or more per day; eating rate on a 5-point scale from very slow to very fast; answer options for eating frequency on work and work-free days were 1–2, 3–4, 5–6, and ≥7 occasions per day.*

Table (suppl) 8: P-values of log-likelihood ratio test for the interaction of night shift exposure and gender on dietary behaviors (age-adjusted, random intercept for country).

|  | *p*-value (interaction night shift x gender) |
| --- | --- |
| Food group intake frequency |  |
| Refined grains | 0.074 |
| Whole grains | 0.827 |
| Legumes and pulses | 0.091 |
| Vegetables | 0.151 |
| Fruits | 0.763 |
| Sugar-sweetened beverages | 0.709 |
| Caffeinated beverages | 0.986 |
| Eating speed | 0.274 |
| Eating frequency on work-free days | 0.269 |
| Eating frequency on work days | 0.484 |

*P-values were derived from log-likelihood ratio tests comparing age-adjusted mixed-effects ordinal logistic regression models with and without an interaction term between night shift exposure (current and former vs. day workers [reference]) and gender, with country included as a random intercept.*

Table (suppl) 9: Gender differences in eating behaviors during night shifts (age-adjusted, random intercept for country)

|  | Men | Women | | |
| --- | --- | --- | --- | --- |
|  | **N=1,937** | **N=1,847** | | |
|  | ***ref.*** | **Odds Ratio** | **95% CI** | |
| Eating frequency during night shifts |  | 1.10 | 0.97 | 1.26 |
|  | **N=1,626** | **N=1,654** | | |
| Timing of eating occasions during night shifts |  |  |  |  |
| At the beginning of night shift |  | 0.87 | 0.74 | 1.01 |
| In the middle of night shift |  | 0.88 | 0.76 | 1.02 |
| At the end of night shift |  | 1.18 | 0.98 | 1.41 |
| It differs per shift |  | **1.42** | **1.20** | **1.69** |
| Determinants of food choice during night shifts |  |  |  |  |
| My appetite |  | 0.99 | 0.86 | 1.14 |
| Time |  | **1.53** | **1.32** | **1.76** |
| Food availability |  | 1.08 | 0.93 | 1.25 |
| My habits |  | **0.73** | **0.62** | **0.85** |
| Energy level |  | **1.72** | **1.44** | **2.04** |
| Mood |  | **1.60** | **1.34** | **1.91** |
| Cost |  | **0.58** | **0.46** | **0.73** |

*Odds ratios (OR) and 95% confidence intervals (CI) were estimated using age-adjusted mixed-effects ordinal logistic regression models comparing women with men (reference), with country included as a random intercept. Items on timing of eating occasions and determinants of food choice allowed multiple responses and were analyzed as separate ordinal outcomes.*
